# Depression literacy and misconceptions scale (DepSter): a new two-factorial tool for measuring beliefs about depression

**DOI:** 10.1186/s12888-023-04796-8

**Published:** 2023-05-01

**Authors:** Katarzyna Kulwicka, Agata Gasiorowska

**Affiliations:** Faculty of Psychology in Wroclaw, SWPS University of Social Sciences and Humanities, Warszawa, Poland

**Keywords:** Beliefs about depression, Depression literacy, Misconceptions about depression, Social perception, Depression

## Abstract

**Background:**

Depression literacy has received extensive attention within mental health research. It has been studied by different social groups and professions in Western and non-Western cultures. The importance of this topic stems from the fact that depression literacy is strongly related to attitudes toward people who are diagnosed with depression, the tendency to stigmatize this mental disorder, and to the propensity to undertake help-seeking behaviors. Therefore, understanding and promoting depression literacy is crucial in contemporary mental health prevention and promotion. We propose a new two-factorial tool measuring beliefs about depression. This 14-item self-report measure captures how people vary across two dimensions of beliefs about depression—depression literacy and misconceptions about depression.

**Methods:**

In ten studies with a total sample of over 4,600 participants from three countries, we demonstrated the two-factorial structure of the Depression Literacy and Misconceptions Scale (DepSter) in Polish (Studies 1 and 2), American (Study 4), and British (Study 5) samples. We showed measurement equivalence for the Polish and English versions of the scale (Study 3). Furthermore, we tested the discriminant meaning of the two dimensions of beliefs about depression analyzing its association with health literacy, mental health literacy, and prejudice toward people with mental illness (Study 4), depression literacy and depression stigma (Study 5), empathetic concerns (Study 7), social dominance orientation (Study 8), and the Big Five personality traits (Study 9). We also investigated whether individuals with formal education in psychology and direct or indirect experience with depression demonstrate a higher level of depression literacy and a lower level of misconceptions about depression (Study 6). Our measure showed high stability for two dimensions of beliefs about depression (Study 10), in both its Polish and English versions, with the measurement conducted after three weeks and three months.

**Discussion:**

We conclude that the proposed approach to beliefs about depression capturing both depression literacy and misconceptions about depression measured with the DepSter scale can easily be applied in clinical and social settings, especially in studies concerning the perception of those diagnosed with depression.

**Supplementary Information:**

The online version contains supplementary material available at 10.1186/s12888-023-04796-8.

## Background

Depression is one of the most common mental disorders, affecting over 264 million people around the globe [1]. It is also the leading cause of disability worldwide and contributes to the overall global burden of disease. Professional, medical knowledge about depression is well-established and systematically growing [2]. Mental health professionals recognize its cognitive, emotional, and behavioral symptoms [3], origins and treatment methods [4, 5], and neurobiological bases and mechanisms [6]. This broad spectrum of professional understanding of depression is based on gradually increasing scientific evidence and therefore, is considered accurate and factual knowledge about depression. However, despite an enormous amount of information about depression derived from scientific knowledge, there are also plenty of lay conceptions—and misconceptions—about this mental health disorder.

Furnham and Telford [7] distinguish three types or concepts within lay conceptions on mental disorders: public attitudes, lay theories, and mental health literacy. Public attitudes refer to the attitudes toward people with mental disorders, beliefs on what they are like (e.g., whether they are dangerous or not), and what kind of treatment they should get [8]. Lay theories focus on lay peoples’ beliefs on the causes and treatment of mental disorders, and the relationship between these two [9, 10]. In other words, the concept of lay theories refers to the way people attribute causes of mental disorders as either biological, psychological, or social sources, and the extent to which appropriate treatment is in line with the nature of the cause of the specific disorder (e.g., to what extent a layperson would perceive taking medications as proper treatment for a disorder that is believed to have biological causes). Although some researchers claim that there is no such thing as “lay” theories because they are always—at least to some extent—derived from scientific theories and conceptions [11], this concept is still studied in mental health research [12, 13].

The third concept, mental health literacy, is crucial for this article. This term was first proposed by Jorm et al. [14] and defined as “knowledge and beliefs about mental disorders which aid their recognition, management, and prevention.” Mental health literacy is considered an individual difference variable and part of more general health literacy [14, 15]. It includes a few components such as knowledge about the development and prevention of mental disorders, treatment methods and their availability, and self-help strategies and skills to support others in a mental health crisis [15]. In recent research, some additional components have been considered part of mental health literacy, such as methods for decreasing stigma related to mental disorders, enhancing help-seeking efficacy, and understanding how to improve and maintain mental health [16]. Furthermore, although mental health literacy refers to knowledge about mental disorders in general, it may also refer to a specific mental disorder such as depression. Hence, depression literacy contains all of the components of mental health literacy, yet in relation to depression [17].

### Depression literacy

As depression is one of the most common mental disorders [1], depression literacy has received extensive attention within mental health research. It has been studied among different social groups and professions in Western and non-Western cultures [18–20]. The importance of this topic stems from the fact that depression literacy is strongly related to attitudes toward people who are diagnosed with depression, and especially to the propensity to stigmatize this mental disorder: better knowledge about depression leads to more positive attitudes toward those with depression and less stigmatization [21, 22]. Depression literacy is also related to the propensity to undertake help-seeking behaviors: the more people know about depression, the more likely it is for them not only to see the need to get professional help, but also to actively seek this kind of help [23, 24]. Therefore, understanding and promoting depression literacy is crucial in contemporary mental health prevention and promotion. However, we believe that, to understand this psychological phenomenon fully, we need to be able to measure it with adequate, reliable, and valid methods.

### Structure and measurement of depression literacy

In a recent systematic review, Singh et al. [25] concluded that the most common approach to measuring depression literacy in adolescents is vignette-based methodology. In this method, participants are presented with a brief description of a person diagnosed with a particular mental disorder and asked to answer several questions measuring depression literacy. These questions usually refer to recognizing the presented disorder, beliefs about treatment, the likelihood of help-seeking, or willingness to assist a person diagnosed with this disorder [14, 26, 27]. The vignette-based approach has several advantages, including simultaneous assessment of multiple components of depression literacy and giving respondents a richer picture than simply referring to “depression,” “mental illness,” or “mentally ill people.” However, one serious drawback of such an approach is the lack of standardization: different authors use different sets of vignettes, making the results of their studies incomparable [28]. Furthermore, the reliability and validity of the interpretation of vignette scores as an indicator of depression literacy are seldom reported, and therefore, the psychometric characteristics of such measures remain unknown.

Another common approach to measuring depression literacy is to employ psychometric scales such as the Depression Literacy Questionnaire (D-Lit) [29, 30] and the Adolescents’ Depression Knowledge Questionnaire (ADKQ) [31], The D-Lit was first developed in Australia to measure this construct in adults, then adapted and validated in different adult populations [32, 33]. It consists of 22 true and false statements on general knowledge about depression, its symptoms, and treatment methods. The participant’s task is to decide whether each statement is true or false. More correct answers indicate a higher level of depression literacy.

The ADKQ [31] assesses depression literacy and its change among youth. It consists of 19 questions, with 15 referring to knowledge about depression and the remaining four questions referring to attitudes toward people with mental disorders. Fourteen questions (13 concerning knowledge and one concerning attitudes) are answered on a dichotomous scale (true/false), while the remaining four are open-ended and require filling the gaps. Unfortunately, this approach makes this scale challenging to use since the participants’ scores need to be calculated using a judge’s evaluation of these open questions. Therefore, the ADKQ seems to be a standardized and validated measure of depression knowledge, but not attitudes toward people with depression.

### A novel approach to depression literacy

Although based on correct (based on medical knowledge) and incorrect (based on lay knowledge) statements about depression, the questionnaires mentioned above construe depression literacy operationalized as depression knowledge as a unidimensional construct, with a higher score on the scale indicating a higher level of depression literacy. Although Hart et al. [31] tested the multifactorial structure of their ADKQ and found that two- and three-factor solutions fitted the data well, the potential dimensions they investigated were related to the knowledge content (depression literacy) and not to its validity: they assumed the existence of factors such as “causes/etiology,” “signs/symptoms,” and “general knowledge” [31]. In this project, however, we propose a novel scale to measure beliefs about depression that reflect both depression literacy operationalized as the knowledge about depression based on medical/psychological evidence and misconceptions about depression operationalized as stereotypical views based on lay theories and culturally driven concepts of what depression is. We assume that one’s beliefs of depression are a mixture of these two components, present to a different extent. High depression literacy means that one has profound and adequate knowledge on the symptoms, causes, and treatment of depression, without sharing beliefs that are relatively common in society but not grounded in scientific knowledge. On the other hand, high levels of misconceptions about depression mean that one’s knowledge is based on stereotypical convictions concerning depression rather than medical/psychological facts. We also assume that a low level of misconceptions about depression might not automatically mean that a person has good depression literacy—such a score might co-exist with a low level of depression literacy if a person did not have any knowledge about depression. Finally, we rooted our model of beliefs about depression in the assumption that a level of depression literacy and a level of misconceptions about depression might have completely different consequences for the well-being of a person and their social surroundings, depending on whether it is associated with a complete lack of knowledge about depression or with sharing beliefs derived from lay theories. In summary, the new tool we present in this article is the first attempt to create a multidimensional measure of beliefs about depression, with dimensions distinguished based on the type of knowledge. Furthermore, the new Depression Literacy and Misconceptions Scale (DepSter) was developed in two language versions and validated in a broad cultural context, making it more applicable to future research.

### The present study

In the remainder of this article, our focus is on validating the two-factorial model of beliefs about depression as measured with DepSter. This scale aims to serve as an integrative measure of beliefs about depression, capturing both a level of depression literacy and a level of misconceptions about depression. To that purpose, we conducted ten studies in which we developed and validated the measure in its Polish and English versions. First, we tested the factorial structure of DepSter and demonstrated its two-factorial structure in Polish (Studies 1 and 2), British (Study 5), and American (Study 4) samples. We demonstrated the measurement equivalence for the Polish and English versions of the scale (Study 3). Going further, we tested the theoretical validity of the scale as well as the convergent and divergent relations of depression literacy and misconceptions about depression , analyzing its association with health literacy, mental health literacy, and prejudice toward people with mental illness (Study 4), depression literacy and depression stigma (Study 5), empathetic concerns (Study 7), social dominance orientation (Study 8), and the Big Five personality traits (Study 9). We also examined the concurrent validity of the scale by investigating whether individuals with formal education in psychology and direct or indirect experience with depression demonstrate a higher level of depression literacy (Study 6). Finally, our measure also showed high test-retest reliability (Study 10), in both the Polish and English versions, with the measurement conducted after three weeks and three months. All studies presented in this manuscript have been accepted by the Ethics Committee at Wroclaw Faculty of Psychology, SWPS University of Social Sciences and Humanities.

For each of the studies (not preregistered), we report how we determined our sample size, all data exclusions, and all measures in the study, and we follow JARS [34]. All data were analyzed using JASP 0.16.4.

## Methods

### Study 1

The aim of Study 1 was to reduce a large pool of items to psychometrically sound ones that would cover two types of beliefs about depression and provide an initial test of the factorial structure of our scale. Before we created the initial item pool, we assumed that DepSter would be a two-factor tool comprising two different aspects of such beliefs, i.e., [1] the level of depression literacy—the level of accurate, professional knowledge stemming from medical and scientific facts about depression; and [2] the level of misconceptions about depression rooted in stereotypes and culturally-based beliefs about depression. The statements comprising depression literacy were derived from the diagnostic criteria of a major depression episode taken from the DSM-V [3] and ICD-10 [35–37], as well as from current scientific knowledge about depression [2]. These statements were formulated in common everyday language rather than the language of medical discourse [38]. For example, “Fatigue or loss of energy nearly every day” [3] was transformed into “Due to depression, people do not have the energy to do anything.” The statements comprising misconceptions about depression were based on data gathered from focus groups on stereotypes about depression conducted with psychology students and the investigation of Internet forums dedicated to this topic. We assumed that our future participants would use a five-point Likert scale indicating to what extent they agreed or disagreed with the statements, as the five-point scale is reported as the most accurate and optimal for such measurements [39]; we formulated all items accordingly. Based on these assumptions, we created a list of 135 statements in Polish, 60 based on accurate (medical and scientific) knowledge, and 75 based on stereotypical (culturally-based) beliefs.

Four independent editors reviewed all items in the next step and provided necessary linguistic corrections. Then, to assess the content validity of the scale, 15 clinical psychologists were provided with the definition and conceptualization of accurate knowledge (depression literacy) and stereotypical knowledge (misconceptions) about depression and asked to evaluate whether our items reflected either of these dimensions using a three-point scale: 2 = “This item is crucial and should be included in the scale,” 1 = “This item represents the construct poorly,” 0 = “Item is not essential and should not be included in the scale” (interrater consistency: ICC = 0.725, 95% CI[0.65, 0.79]). Finally, we chose items evaluated as “2” by 12 or more out of the 15 judges (CVR index of 0.60 or higher). The preliminary version of the scale consisted of 15 items, six representing depression literacy and nine misconceptions about depression.

Using this pool, we collected data to initially test a measurement model that grouped the 15 items into two first-order factors representing depression literacy and misconceptions about depression. We expected that this model would have a better fit than the model where all 15 items would converge into one factor, without distinguishing separate dimensions for depression literacy and misconceptions about depression.

### Participants and procedure

We calculated our sample size using the calculator proposed by Preacher and Coffman [40]. We assumed a very conservative scenario, that our items are relatively independent and hence, the RMSEA for the null model would be low (0.12), and that the alternative RMSEA for the two-dimensional model would be at the highest value for acceptable fit (0.08). With such assumptions, we found that a sample of 250 participants would allow for results significant at 0.001 with a power of 0.95. Hence, we decided that in this initial study we would double this number and recruit at least 500 participants.

Five hundred and seventy-six Polish volunteers (see Table 1 for details of all samples), participated in an online study without compensation. Participants were recruited via a snowball sampling technique. After providing informed consent, the participants’ task was to answer demographic questions and complete a 15-item version of DepSter as a part of a larger study. For each item, they indicated their agreement on a Likert scale ranging from 1 = “Strongly disagree” to 5 = “Strongly agree.” No data were discarded.

**Table 1:** Participants demographics in Studies 1–10b

Study	Country			Gender		Age		
		Before exclusions	Final	W	M	range	M	SD
1	PL	576	576	473	103	15–87	33.05	11.17
2	PL	817	798	417	382	21–81	44.98	14.40
3	PL	207	195	127	68	17–65	31.70	8.88
4	US	318	271	117	154	20–71	36.15	10.99
5	UK	603	590	390	200	18–77	36.88	12.72
6	PL	411	411	345	66	20–87	34.05	10.45
7	PL	587	587	478	109	15–71	33.90	9.84
8 a	UK	401	389	263	126	18–69	34.62	11.82
8b	PL	394	394	323	71	18–71	28.38	9.11
9	PL	364	364	289	75	15–87	35.82	12.20
10 a	US	112	112	54	58	20–69	37.47	10.85
10 b	PL	123	123	99	24	15–75	31.91	11.36

## Results

Firstly, we conducted a confirmatory factor analysis (CFA) with the maximum likelihood estimation method and robust estimation of standard errors. This procedure takes the non-normality of outcomes into account. We tested the proposed model of the DepSter scale, grouping the initial 15 items into two first-order factors representing depression literacy and misconceptions about depression. The CFA yielded a good fit for this model in light of some but not all fit indices (see Table 2). Although all standardized factor loadings were significant at p < .001, the value for one item from the misconceptions about the depression dimension (“Seeing a psychologist or a psychiatrist in order to receive help with fighting depression is a sign of weakness”) was very low (β = 0.194), leading us to remove this item from the scale. As a result, we were left with a 14-item scale, which again exhibited a similar data fit.^1^ Standardized factor loadings for all items were significant at p < .001. The standardized factor loadings and item-dimension correlations for the 14 DepSter items are presented in Table 3. All the item-dimension correlations exceeded 0.20, confirming the discriminative power of the 14 items.

**Table 2:** Model fit indices for the two-factor model and one-factor model of the DepSter scale in Studies 1–5

Study	Two-factor model						One-factor model						Δχ^2^/Δdf
	χ^2^/df	RMSEA	90%CI	SRMR	TLI	CFI	χ^2^/df	RMSEA	90%CI	SRMR	TLI	CFI	
1 ver1	4.15	0.074	[0.066, 0.082]	0.060	0.84	0.86	5.72	0.091	[0.083, 0.098]	0.069	0.79	0.73	145.23*
1 ver2	4.24	0.075	[0.067, 0.084]	0.060	0.85	0.88	6.11	0.094	[0.086, 0.102]	0.071	0.80	0.76	147.93*
2	4.94	0.071	[0.063, 0.079]	0.046	0.93	0.94	18.70	0.150	[0.142, 0.157]	0.104	0.67	0.73	899.40*
4	3.85	0.103	[0.090, 0.115]	0.072	0.87	0.89	4.16	0.108	[0.096, 0.120]	0.074	0.85	0.87	27.43*
5	3.84	0.069	[0.061, 0.078]	0.061	0.88	0.90	4.44	0.076	[0.068, 0.085]	0.062	0.86	0.88	49.42*

NOTE: ver1: 15 item model, ver2: 14 item model, Δdf = 1, * *p* < .001

**Table 3:** Standardized factor loadings and item-dimension correlations for the DepSter items (Study 1)

Item	Standardized factor loadings	Item-rest correlations
Depression Literacy (DL)		
1. Depression is an illness	0.45***	0.32
2. Depression may affect anyone	0.43***	0.33
3. Depression makes people lose interest even in the things they used to enjoy doing	0.54***	0.43
4. Depression makes people lack the strength to do anything	0.54***	0.42
5. People with depression often think about suicide	0.43***	0.36
6. Depression is associated with great suffering	0.56***	0.41
Misconceptions about depression (MiscD)		
7. Depression is just a fad	0.56**	0.50
8. Depression is just a temporary mood deterioration	0.35**	0.34
9. Depression affects only people who are weak and cannot cope with their life	0.52***	0.51
10. To overcome depression, all you need is willpower	0.86***	0.68
11. To overcome depression, all you need is to get yourself together	0.88***	0.70
12. Antidepression medication starts to work right after intake	0.27***	0.27
13. Depression is just self-pity	0.57***	0.53
14. People with depression are mentally weak	0.45***	0.47

* p < .05, **p < .01, ***p < .001

Secondly, we also tested the alternative, one-dimensional structure of the DepSter scale, assuming that all the items load into just one factor representing depression literacy. This model fit was worse than for the previous model. Again, factor loading for one item was much lower than the other factor loadings. After excluding this item from the scale, the model was still worse than the respective two-factor model (Table 2).

In sum, we decided to conclude on the two-factor structure of the scale, as it was better fitted and more relevant to our theoretical approach. In other words, beliefs about depression measured with the DepSter scale consist of having a level of depression literacy and a level of misconceptions about depression. Moreover, we decided to exclude the one item with the lowest factor loading in all analyses and use the 14-item version of the scale.

Regarding its readability, the final scale had a satisfying Gunning fog index of 4.56, meaning that it would be easily understood by somebody with about one to six years of formal education. We also evaluated DepSter’s internal consistency. Cronbach’s α was slightly below the conventional threshold for the depression literacy subscale (α = 0.64) and satisfactory for the misconceptions about depression subscale (α = 0.78).

### Study 2

Having garnered initial support for the proposed structure of beliefs about depression as measured with DepSter in Study 1, the aims of Study 2 were threefold: [1] to confirm the adequacy of the scale in a representative sample of adult Poles; [2] to inspect if the scale yielded any age, gender, or education differences; and [3] to establish the initial validity and reliability of the scale.

According to research on general health literacy and mental health literacy, levels of these two constructs differ among people in different age groups. In general, older people have less adequate and less accurate knowledge about physical diseases, their causes, and treatment methods than younger subjects [16, 31, 43]. The same pattern is observed for mental disorders [44, 45]. Moreover, older age is also a predictor of more negative attitudes toward people diagnosed with mental disorders [46, 47]. Therefore, we expected a positive correlation between age and misconceptions about depression and a negative correlation between age and depression literacy.

Women are more likely than men to engage in healthy lifestyle choices and health-related behaviors [48, 49], as well as health information seeking [50]. They also present greater general health-related knowledge than men [51] and greater mental health literacy [52]. Furthermore, a recent review of the concept of mental health literacy, its correlates, and importance revealed that female gender is also one of the most important predictors of mental health literacy [53], since the vast majority of reviewed studies reported that women have better knowledge about mental disorders and are more accurate in recognizing symptoms of them than men. Therefore, we expected that women would demonstrate lower scores on misconceptions about depression and higher scores on depression literacy than men. Moreover, we hypothesized that less educated people would score higher on misconceptions about depression and lower on depression literacy than more educated people. We rooted this expectation in the results showing that people with a better education present a better understanding of mental disorders and better accuracy in the recognition of symptoms [53, 54].

### Participants and procedure

In this study, we decided to double the sample size that we calculated for Study 1. Hence, N = 817 participants, constituting a representative sample of Polish adults in terms of gender, age, education, and residence, contributed to an online study with compensation provided by the Ariadna Internet research panel. After providing informed consent, the participant’s task was to answer demographic questions and complete a 14-item version of DepSter as a part of a larger study. The order of items in the DepSter scale was randomized. We embedded two questions in the survey, serving as attention checks. In the first one, embedded in the demographic section, we asked participants to provide the current year. In the second one, embedded in the DepSter scale, we asked them to mark a specific answer (“strongly agree”). Based on the attention checks, 28 participants were excluded from further analysis (eight did not provide a valid year, and another 20 did not mark the appropriate answer). The final sample consisted of N = 789 participants (Table 1). Concerning the level of education, 12 participants indicated Level 1^2^ (1.5%), 8 participants declared Level 2, 102 participants declared Level 3, 357 participants declared Level 4, and 310 participants declared Level 6 and higher.

### Results

#### Internal validity

Firstly, we retested the proposed model of the scale using CFA with the maximum likelihood estimation method and robust estimation of standard errors. To reiterate, this model groups the 14 items into two first-order factors representing two dimensions of depression literacy and misconceptions about depression. The CFA yielded a good fit for this model (Table 2), and standardized factor loadings for all items were significant at p < .001. As in Study 1, we also tested the alternative, one-dimensional structure of the DepSter scale, assuming that all items load into just one factor representing beliefs about depression. Again, this model fit was worse than for the previous model (Table 2). Both dimensions had good internal consistency (α = 0.84 for DL and 0.86 for MiscD), and the two dimensions correlated negatively, r = − 0.45, p < .001. Overall, these results provide additional evidence for the good internal validity of the bi-dimensional structure of the scale in a more heterogeneous sample of Polish participants.

#### External validity

Contrary to what we expected, we did not observe significant correlations between age and depression literacy, r(789) = − 0.002; p = .965, nor misconceptions about depression, r(789) = 0.02; p = .645. As hypothesized, women scored higher on depression literacy (M = 4.24, SD = 0.61) than men (M = 3.93, SD = 0.67), t(752.622) = 6.65, p < .001, Cohen’s d = 0.47, while, men scored higher on misconceptions about depression (M = 2.49, SD = 0.79) than women (M = 2.07, SD = 0.74), t(787) = − 7.62; p < .001, Cohen’s d = 0.55. Finally, as expected, the level of participants’ education was positively correlated with depression literacy, Spearman’s Rho = 0.11; p = .001, while it negatively correlated with misconceptions about depression, Spearman’s Rho = − 0.21; p < .001.

To sum up, the results of Study 2 confirmed the proposed structure of DepSter and its psychometric adequacy in a second, representative sample of Polish adults, and allowed us to establish the basic demographic patterns of the scale, which revealed no age effect and small-to-moderate gender and education effects, with relatively better-educated participants and women scoring slightly higher on depression literacy, while scoring lower on misconceptions about depression. Finally, we demonstrated that the DepSter’s subscales have good reliability, measured as internal consistency.

### Study 3

This study aimed at developing an English version of DepSter using a back-translation procedure and testing the equivalence of the two language versions. Firstly, 14 items were translated from Polish into English by a professional translator who consulted with native English speakers. Next, another professional translator translated these items from English into Polish. Secondly, we compared two Polish versions and made the necessary corrections in the English version of the items. Finally, we asked participants fluent in English and Polish to fill out both versions of the scale and tested correlations between these two versions and their internal consistencies.

### Participants and procedure

Since in this study we investigated the correlations between two language versions of the scale, we calculated our sample size assuming that we wanted it to be large enough to detect a correlation of 0.3 at a significance of 0.01 with a power greater than 0.95, giving us a sample size of 182 participants [56]. Factoring for potential attrition due to the lack of language proficiency, we invited 207 Polish participants to complete both the English and Polish versions of DepSter in an online study. Participants were also asked to assess their language proficiency (for both English and Polish) on a nine-point scale from 1 = “Beginner” to 9 = “Very advanced” and answer one question on the current year serving as an attention check (the same as in Study 2). Their participation was not rewarded. We excluded 11 participants from further analysis (five were excluded based on insufficient language proficiency, and six failed the attention check). The final sample consisted of 195 participants (Table 1).

### Results

The correlations between the dimensions and total score of the Polish and English versions of DepSter with their respective internal consistencies are presented in Table 4. For the Polish version of the scale, Cronbach’s α was slightly below the conventional threshold for the depression literacy dimension, but high for the misconceptions about depression subscale. For the English version of the scale, Cronbach’s α was high for both dimensions.

**Table 4:** Internal consistency, descriptive statistics, and intercorrelations for the two language versions of the DepSter subscales in Study 3 (N = 196)

Dimension	α	M	SD	1.	2.	3.
Polish version						
1. Depression Literacy (DL)	0.61	4.38	0.44	-		
2. Misconceptions about Depression (MiscD)	0.83	1.54	0.55	−0.51**	-	
English version						
3. Depression Literacy (DL)	0.76	4.30	0.61	0.70**	−0.37**	-
4. Misconceptions about Depression (MiscD)	0.84	1.58	0.61	−0.33**	0.71**	−0.46**

* p < .05, **p < .01, ***p < .001

As expected, we observed high correlations between the scores on the depression literacy and misconceptions about depression subscales in both language versions (Table 4). The pattern of correlations between the two dimensions was similar across language versions, respectively Z = 1.51, p = .132 for the comparison of correlations between depression literacy and misconceptions about depression.

To conclude, the results of Study 3 demonstrated the similarity of the Polish and English versions of DepSter, together with their satisfactory internal consistency.

### Study 4

This study aimed to confirm the structure of the DepSter scale in an American sample and provide an initial test of the convergent and divergent validity of the scale’s score interpretation and the discriminant validity of the two factors of DepSter. We investigated the relationship between beliefs about depression measured with our scale and other constructs such as health literacy, mental health literacy, and prejudice toward people diagnosed with mental disorders. We started DepSter validation by examining its relationship with health and mental health literacy, since these two constructs represent a similar theoretical field as our scale. Health literacy is a concept that reflects peoples’ ability and motivation to seek health information to maintain good health [43, 57]. A high level of health literacy is associated with better knowledge about chronic diseases [58], better health status [59], and even a lower mortality rate among older adults [43]. Crucially, from our point of view, a high level of health literacy is also related to a better understanding of mental disorders and with better mental health in general [60]. Therefore, we hypothesized that scores on the depression literacy subscale should be positively correlated with scores on the scale measuring health literacy. We also expected that the misconceptions about depression subscale and health literacy scale should be negatively correlated. However, the strength of this correlation should be lower than for the depression literacy subscale.

As described in the introduction, mental health literacy is a subtype of health literacy encompassing knowledge and beliefs about mental disorders, the ability to recognize symptoms of mental disorders, knowledge about methods of treatment, and self-help strategies [14, 15] but also on strategies to reduce mental health-related stigma [16]. Hence, we hypothesized that depression literacy should positively correlate with mental health literacy, while misconceptions about depression should correlate negatively. Furthermore, as mental health literacy is conceptually closer to depression literacy than general health literacy, we expected these correlations to be stronger than correlations between DepSter’s subscales and the health literacy measure.

As a low level of mental health literacy is associated with more negative attitudes and greater prejudice toward people with mental illness [61], we tested the relationship between the two dimensions of DepSter and the level of prejudice mentioned above. We expected that depression literacy would negatively correlate with such prejudice, while we expected an inverse relationship with misconceptions about depression. Furthermore, we expected that the relationship between misconceptions about depression and prejudice would be stronger than the relation for depression literacy. Again, as prejudice toward people with mental illness is conceptually closer to depression literacy than general health literacy, we expected these correlations to be stronger than the correlations between DepSter subscales and the health literacy measure.

### Participants and procedure

In this study, we aimed to investigate the factorial structure of the English version of DepSter using CFA. Concerning the sample size, we relied on the power analysis we conducted for Study 1, and aimed to recruit at least 250 participants in this study. Factoring for potential attrition due to attention checks, we recruited 318 US residents to take part in the study conducted via the Amazon Mechanical Turk platform in exchange for $1.30. After providing informed consent, their task was to fill out an English version of DepSter and questionnaires measuring the level of health literacy, mental health literacy, and prejudice toward people with mental illness. The questionnaires were presented in random order. We also embedded the same two questions that served as an attention check in Study 2 and excluded 46 participants from the analysis, leaving a sample of N = 271 participants (Table 1). The sample was large enough to detect a correlation of 0.24 at p = .01 with 0.95 power.

We measured health literacy with the Health Literacy Survey (HLS-Q6) [62]. The HLS-Q6 consists of six items such as “On a scale from very easy to very difficult, how would you say it is to use information the doctor gives you to make decisions about your illness” or “On a scale from very easy to very difficult, how would you say it is to find information on how to manage mental health problems like stress or depression” (α = 0.80). Participants indicated their answers using a four-point scale (1 = “Very difficult” to 4 = “Very easy”), with a higher score indicating a higher level of health literacy.

To measure mental health literacy, we used the Mental Health Literacy Scale (MHLS) [63], assessing attributes of mental health literacy such as the ability to recognize mental disorders, knowledge of how to seek mental health information, and attitudes that promote recognition and appropriate help-seeking. It consists of 35 items (α = 0.87). On the first 15 items, participants indicated their answers using a four-point scale (1 = “Very unlikely” to 4 = “Very likely”), such as “If someone experienced a low mood for two or more weeks, had a loss of pleasure or interest in their normal activities, and experienced changes in their appetite and sleep, then to what extent do you think it is likely they have a major depressive disorder.” On 16 items (e.g., “I am confident that I know where to seek information about mental illness”), participants indicated their answers using a four-point scale (1 = “Strongly disagree” to 4 = “Strongly agree”). On seven items (e.g., “How willing would you be to move next door to someone with a mental illness”), participants indicated their answers using a four-point scale (1 = “Definitely unwilling” to 4 = “Definitely willing”).

To measure prejudice toward people diagnosed with mental disorders, we employed the Prejudice towards People with Mental Illness scale (PPMI) [64]. It consists of 28 items such as “I would find it hard to talk to someone who has a mental illness,” “People who are mentally ill are avoiding the difficulties of everyday life,” or “The behavior of people with mental illness is unpredictable.” Participants indicated their answers on a nine-point scale ranging from − 4 (“Very strongly disagree”) to + 4 (“Very strongly agree”), with a higher score reflecting a greater level of prejudice (α = 0.91).

### Results

#### Internal validity

As a first step, we retested the proposed model of the scale in its English version. To reiterate, this model grouped the 14 items into two factors: depression literacy and misconceptions about depression. A maximum likelihood CFA demonstrated that the fit for this model was worse than in the Polish sample (Table 2), possibly be due to the smaller sample size. As in previous studies, we also tested the alternative, one-dimensional structure of the DepSter scale, assuming that all items loaded into just one factor representing beliefs about depression. Again, this model fit was worse than for the previous model (Table 2). These results provide additional evidence for the bi-dimensional structure of the scale, this time in its English version, and its good internal validity. Cronbach’s α for the depression literacy dimension was below the conventional threshold (α = 0.59), while it was high for misconceptions about depression (α = 0.93).

#### Convergent and divergent validity

The correlations between DepSter and other used measures are presented in Table 5.

**Table 5:** Correlations between the scores on DepSter subscales and other constructs in Studies 4–9

Measures	DL	MiscD
Study 4 (N = 271)		
Health literacy (HLS-Q6)	0.12*	−0.06
Mental health literacy (MHLS)	0.43**	−0.67**
Prejudice towards people with mental illness (PPMI)	−0.32**	0.64**
Study 5 (N = 590)		
Depression literacy (D-Lit)	0.14**	−0.40**
Depression stigma	0.06	0.44**
Personal stigma	−0.06	0.56**
Perceived stigma	0.15**	0.17**
Study 7 (N = 587)		
Empathetic concern	0.34**	−0.14**
Perspective taking	0.24**	−0.19**
Personal distress	−0.01	−0.09*
Empathetic sensitivity	0.29**	−0.20**
Study 8		
Social dominance orientation, UK sample (N = 389)	−0.19**	0.54***
Social dominance orientation, Polish sample (N = 394)	−0.09	0.26**
Study 9 (N = 364)		
Neuroticism (TIPI)	−0.19**	0.19**
Extraversion (TIPI)	−0.05	0.02
Openness (TIPI)	0.01	−0.15**
Agreeableness (TIPI)	−0.07	0.05
Conscientiousness (TIPI)	−0.03	−0.03

* p < .05, **p < .01, ***p < .001

In line with our prediction, depression literacy was positively correlated with health literacy and mental health literacy and negatively correlated with prejudice toward people with mental illness. Again, in line with our expectations, misconceptions about depression was negatively correlated with mental health literacy and positively correlated with prejudice toward people with mental illness. However, contrary to our expectations, it was not associated with health literacy.

Confirming our expectations, DepSter’s subscales correlated with mental health literacy and prejudice toward people with mental illness more strongly than with general health literacy. Furthermore, although we failed to demonstrate that misconceptions about depression is negatively related to health literacy, we confirmed that the correlation between health literacy and depression literacy is stronger than between the former and stereotypical knowledge. This pattern possibly stems from the fact that the HLS-Q6 focuses mainly on the ability to seek accurate information about health, disease prevention, and health promotion [62] and not on general knowledge about health-related issues. Finally, we expected that prejudice toward people with a mental illness would correlate more strongly with misconceptions about depression than depression literacy, which is precisely what we found.

To sum up, the results of Study 4 again corroborated the structure of DepSter, this time in an American sample, and provided an initial confirmation of the theoretical validity of the scale.

### Study 5

The aim of this study was twofold: to reconfirm the structure of DepSter in its English version in a different population and a larger sample, and to demonstrate its convergent and divergent validity [65] by analyzing correlations with another popular measure of depression literacy as well as with the Depression Stigma Scale [29].

Depression literacy is a form of mental health literacy defined as “the knowledge and beliefs about mental disorder which aid their recognition, management, or prevention” [14]. More recently, the definition was extended to knowledge on whether or not the mental disorder is developing, knowledge about treatment availability and self-help strategies [15], and comprehension of how to decrease mental health stigma [16]. One of the first and most commonly used tools to assess depression literacy (D-Lit) was developed by Griffiths et al. [29]. Therefore, we hypothesized that if DepSter is valid, the score on the depression literacy subscale should positively correlate with the score on D-Lit and that the score on the misconceptions about depression subscale should correlate negatively with the score on D-Lit, with the former correlation being stronger than the latter. We further hypothesized that misconceptions about depression would be positively related to depression stigma and the personal stigma subscale, while depression literacy measured with DepSter would negatively correlate with these two constructs. As depression literacy does not reflect the beliefs about other people’s opinions on depression [14–16], we expected, at most, modest correlations between perceived stigma and the score on DepSter.

### Participants and procedure

In this study, we again aimed to investigate the factorial structure of the beliefs about the depression model using CFA as well as the relations between depression literacy and misconceptions about depression with relevant constructs using correlation analysis. Concerning the sample size, we again relied on the power analysis we conducted for Study 1, but this time aimed to double it and recruit at least 500 participants. Factoring for potential attrition due to attention checks, we recruited 603 Prolific Academic users from the UK who took part in an online study in exchange for £0.60. As 13 participants did not provide proper answers to the attention checks (the same as in previous studies), they were excluded from the analysis, leaving a sample of N = 590 participants (Table 1). The sample was large enough to detect a correlation of 0.17 at p = .01 with 0.95 power.

After providing informed consent, participants were asked to fill out an English version of DepSter, the D-Lit questionnaire, and the Depression Stigma Scale [29]. The order of questionnaires and the order of items within the questionnaires were randomized.

D-Lit [29] consists of 22 statements about depression (both correct and incorrect), constituting one dimension. The task of the participant is to mark whether a statement is true or false (e.g., “People with depression often speak in a rambling and disjointed way” or “Sleeping too much or too little may be a sign of depression”). The number of correctly marked statements indicates the participant’s depression literacy (α = 0.70).

Depression stigma, measured with the Depression Stigma Scale [29], reflects one’s attitudes toward people diagnosed with depression (α = 0.78). It consists of 18 items constituting two dimensions that measure: [1] personal stigma (α = 0.76) that is, participants’ attitudes toward people diagnosed with depression (e.g., “Depression is a sign of personal weakness,” “If I had depression I would not tell anyone,” and “I would not employ someone if I knew they had been depressed”); and [2] perceived stigma (α = 0.82), i.e., the participant’s beliefs about the attitudes of others toward people diagnosed with depression (e.g., “Most people believe that depression is a sign of personal weakness,” “If they had depression, most people would not tell anyone,” and “Most people would not employ someone they knew had been depressed). Participants provided their answers on a five-point scale from 1 = “Strongly disagree” to 5 = “Strongly agree”.

### Results

#### Internal validity

As a first step, since we collected data from a new (British) population, we retested the proposed two-factor model of the scale. A CFA with maximum likelihood estimation with robust errors yielded a good fit for this model in light of most fit indices. As in previous studies, we also tested the alternative, one-dimensional structure of the DepSter scale. Its fit was slightly worse than for the default model (Table 2). Cronbach’s α was low for depression literacy (α = 0.49) and high for misconceptions about depression (α = 0.85).

#### Convergent and divergent validity

The correlations between DepSter, D-Lit, and the Depression Stigma Scale are presented in Table 4. As predicted, the score on the depression literacy dimension correlated positively, yet low, with the score on the other measure of depression literacy (D-Lit), while the score on the misconceptions about depression dimension correlated negatively with the score on D-Lit. The low correlation of depression literacy measured by the DLS and this same construct measured by D-Lit may result from the specificity of the latter. Although D-Lit consists of accurate and inaccurate statements about depression, it also includes questions about the symptoms of other mental disorders that can be confused with depression. In contrast, DepSter focuses solely on the symptoms of depression. To some extent, D-Lit may therefore be considered as a somehow more general measure of mental health literacy, not depression literacy itself when compared to DepSter’s depression literacy subscale.

As predicted, the level of misconceptions about depression correlated positively with these two constructs. We did not observe a correlation between depression literacy and depression stigma or between the level of depression and personal stigma. The level of perceived stigma turned out to weakly positively correlate with both the level of misconceptions about depression and depression literacy.

Overall, the results of Study 5 again corroborated the structure of DepSter, this time in a British sample, and provided further confirmation of the theoretical validity of the scale.

### Study 6

So far, we have demonstrated the relationship between depression literacy and misconceptions about depression measured with DepSter and other constructs from the same domain (general or specific health literacy). The aim of Study 6 was to establish the criterion validity of the DepSter subscales. We predicted that depression literacy would be higher for participants who have formal education in psychology and those who have previous experience with depression, either being diagnosed themselves or being familiar with a person diagnosed with depression. On the contrary, the level of misconceptions about depression would be lower for participants who have formal education in psychology and those who have previous experience with depression, either being diagnosed themselves or being familiar with a person diagnosed with depression.

Previous studies have demonstrated that the representatives of different mental health professions differ in levels of mental health literacy. For example, psychiatrists have higher literacy levels than psychiatric nurses [66], while psychologists are more accurate in recognizing symptoms of mental disorders than counselors [67]. Regardless of these differences among mental health professionals, they have more accurate knowledge about mental disorders than non-mental health professionals [68, 69] and the general public. Therefore, we expected that DLS scores would differ depending on whether participants had formal education in psychology. We presumed that those with a university degree in psychology would score lower on misconceptions about depression but higher on depression literacy compared to those educated in different disciplines.

Recent research has also revealed that previous contact with a person diagnosed with a mental disorder predicts mental health literacy [54, 70]. For example, people who have experienced depression were more likely to recognize its symptoms than those who have not been diagnosed with it [71], indicating that they might have more accurate knowledge of the disease. Similarly, people familiar with a person diagnosed with depression recognized depressive symptoms more accurately than those who did not know a person with such a diagnosis [72]. In line with these results, we expected the highest scores on depression literacy for people who suffered from depression, moderate scores for people familiar with a diagnosed person but who did not suffer from it, and lowest scores for those without previous contact with depressed people. We expected the reversed pattern for the misconceptions about depression.

### Participants and procedure

We had no strict prediction for the effect size in comparison between our three groups, so we assumed that the sample size should be large enough to detect η^2^ = 0.05. A priori power analysis [56] revealed that we would need a sample of at least 399 participants to detect such an effect at p = .01 with a power greater than 0.95.

Since, as we demonstrated in Study 2, education level might also play a role in depression literacy, we invited only participants who had university degrees to exclude level of education as a potential confounder. Four hundred and eleven Polish participants (Table 1) participated in an online study without compensation. In addition to filling out the DepSter scale, they provided information on the type of their education that we further coded as psychology (144 participants, 35% of the sample) or other (267, 65% of the sample). They were also asked if they had ever been diagnosed with depression themselves and if they were familiar with a person who had ever been diagnosed with depression. One hundred and eleven participants (27% of the sample) declared that they had been diagnosed with depression, and 221 participants (53.8% of the sample) declared that they were familiar with a person who had been diagnosed with depression. The remaining 79 participants (19.2% of the sample) declared that they had not been diagnosed with depression themselves and were not familiar with a person who had been diagnosed with depression. Cronbach’s α was satisfactory for the depression literacy subscale (α = 0.66) and high for the misconceptions about depression subscale (α = 0.80).

### Results

In line with our expectations, we observed the predicted difference between psychology graduates and others concerning the level of misconceptions about depression, t(398.97) = 6.01, p < .001, Cohen’s d = 0.57, as the former group scored lower (M = 1.32, SD = 0.38) than the latter (M = 1.61, SD = 0.60). The difference between the two groups concerning the level of depression literacy was not significant, t(409) = − 1.27, p = .204, Cohen’s d = 0.12.

Further analysis revealed that the three groups of participants who differed in their depression experience also differed in the level of depression literacy, F(2, 175.06) = 5.44, p = .005, ηp^2^ = 0.033, and misconceptions about depression F(2, 175.38) = 15.17, p < .001, ηp^2^ = 0.090. Notably, these differences were stronger in terms of effect sizes for misconceptions about depression than for depression literacy. Further post hoc tests using Games-Howell’s correction showed that people who had no previous contact with depression demonstrated lower levels of depression literacy (M = 4.37, SD = 0.52) than participants who had been diagnosed with depression themselves (M = 4.60, SD = 0.43), t(146.49) = 3.29, p = .004, Cohen’s d = 0.54, and than participants who were familiar with a person who had been diagnosed with depression (M = 4.54, SD = 0.41), t(113.95) = 2.66, p = .024, Cohen’s d = 0.39. However, participants who were familiar with a person diagnosed with depression or who had been diagnosed with this disorder themselves did not differ with regards to depression literacy, t(211.87) = 1.27, Cohen’s d = 0.14. Moreover, participants who had no previous contact with depression demonstrated higher levels of misconceptions about depression (M = 1.82, SD = 0.74) than participants who knew a person who had been diagnosed with depression (M = 1.49; SD = 0.48), t(102.73) = 3.66, p = .001, Cohen’s d = 0.63, and those who had been diagnosed with depression themselves, (M = 1.34, SD = 0.30), t(109.61) = 5.29, p < .001, Cohen’s d = 0.92. Finally, participants who knew a person who had been diagnosed with depression demonstrated slightly higher levels of misconceptions about depression than participants who had been diagnosed with depression themselves, t(263.23) = 2.56, p = .005, Cohen’s d = 0.30.

To sum up, this study provided further support for the criterion validity of the DepSter scale by evaluating whether formal psychological education and previous contact with depression resulted in higher levels of depression literacy and lower levels of misconceptions about this disorder.

### Study 7

The aim of Study 7 was to further investigate the convergent validity of DepSter subscales [65]. For this purpose, a large sample of Polish participants was asked to complete DepSter together with a measure of empathy, a construct that we expected to be related to depression literacy and misconceptions about depression.

Empathy is defined as a construct describing one’s reactions to other people’s experiences [73]. It manifests as the attempt to take others’ perspectives while observing them in difficult situations [74]. Empathy is crucial for other people’s perception [75–77]. Not surprisingly, empathy is also considered one of the crucial factors for a better understanding of those suffering from mental illness or other health-related problems [61, 78]. Hence, we expected empathetic concern and perspective taking to be positively related to depression literacy and negatively associated with misconceptions about depression. We also expected that depression literacy and misconceptions about depression would not be related to personal distress.

### Participants and procedure

In this study, we expected that the correlation between misconceptions about depression and empathetic concerns might be relatively weak, hence we calculated our sample size assuming that we wanted it to be large enough to detect a correlation of 0.2 at a significance of 0.01 with a power greater than 0.95, which gave us a sample size of 431 participants [56].

Five hundred and eighty-seven Polish residents (478 women, 109 men, aged 15–71 years, M = 33.90; SD = 9.84) participated in the online study without compensation. The link to the study was distributed via multiple web pages, including social media platforms. Participants filled out the Empathetic Sensitivity Scale [79] along with DepSter. The order of questionnaires was randomized. Cronbach’s α was high for both depression literacy (α = 0.92) and misconceptions about depression subscales (α = 0.84).

The Empathetic Sensitivity Scale is a modified version of the Interpersonal Reactivity Index [73] and consists of 28 items grouped into three subscales. The empathetic concern subscale (α = 0.78) consists of 11 items (e.g., “I would describe myself as a pretty soft-hearted person” or “I am often quite touched by things I see happen”) and measures one’s ability to have compassion toward others. The perspective taking subscale (α = 0.74) consists of nine items (e.g., “I try to look at everybody’s side of a disagreement before I make a judgment” or “I sometimes try to understand my friends better by imagining how things look like from their perspective”) and measures one’s ability to consider someone else’s point of view. Finally, the third subscale, personal distress (α = 0.78), measures the feeling of distress while observing other people’s suffering and struggle, and consists of eight items (e.g., “I sometimes feel helpless when I am in the middle of a very emotional situation” or “Being in a tense emotional situation scares me”). Participants ranked their answers on a five-point scale ranging from 1 = “Totally disagree” to 5 = “Totally agree”.

### Results

The results are presented in Table 4. As hypothesized, the score on depression literacy was positively correlated with the scores on empathetic concern and perspective taking, while scores on the misconceptions about depression subscale negatively correlated with the aforementioned subscales of the Empathetic Sensitivity Scale. Moreover, the score on the depression literacy subscale did not correlate with the score on personal distance. However, we observed a significant yet small correlation between the misconceptions about depression subscale scores and the personal distress subscale score.

Overall, Study 7 confirmed the theoretical validity of the DepSter scale.

### Study 8

The aim of Study 8 was to demonstrate the similar validity of DepSter in two different cultures. For this purpose, a large sample of British participants completed the DepSter scale together with the Social Dominance Orientation Scale [80]. Furthermore, we tested whether a similar correlation pattern would also occur in a Polish sample.

Social dominance expresses the level of preference toward social distance [80]. People with a high social dominance orientation prefer greater interpersonal distance than those who score low in social dominance [81]. A high level of this variable is related to prejudice and legitimizing different types of myths about the members of an out-group [82, 83]. This construct also plays a crucial role in stereotyping: those high in social dominance are more eager to use stereotypes in their judgment about other people and are less likely to change this stereotypical point of view [84, 85]. Furthermore, what is crucial for us is that social dominance orientation is related to using stereotypes and the willingness to maintain a distance from people diagnosed with mental illness [86]. For that reason, we expected scores on the Social Dominance Orientation Scale to correlate negatively with scores on the depression literacy subscale and positively with scores on the misconceptions about depression scale, with the latter being stronger than the former. We assumed that a similar correlation pattern in the British and Polish samples would speak in favor of the theoretical validity of our scale.

### Participants and procedure

In this study, we expected that the correlation between depression literacy and social dominance orientation might be relatively weak, hence we calculated our sample size assuming that we wanted it to be large enough to detect a correlation of 0.2 at p = .01 with a power greater than 0.95, giving us a sample size of 431 participants [56]. Due to financial constraints, concerning the British sample, we recruited N = 401 Prolific Academic users from the UK (269 women and 132 men, aged 18–69 years, M = 35.35, SD = 17.67) to participate in this study in exchange for £0.60. Twelve participants were excluded based on two attention checks (the same as in previous studies), and the final analysis was conducted on the data from 389 participants (263 women, 126 men, aged 18–69 years, M = 34.62, SD = 11.82). Concerning the Polish sample, we recruited 394 psychology students from Poland (323 women, 71 men, aged 18–71 years, M = 28.38, SD = 9.11) to participate in the online study in exchange for credit points.

Participants filled out the DepSter scale and the Social Dominance Orientation (SDO) Scale [80, 87]. The order of questionnaires and the order of items within questionnaires were randomized. Cronbach’s α for the DepSter scale was acceptable for the depression literacy subscale (α = 0.52 in the UK sample and α = 0.78 in the Polish sample) and high for the misconceptions about depression subscale (α = 0.84 in the UK sample and α = 0.78 in the Polish sample).

The SDO scale (α = 0.91 for the UK sample, α = 0.91 for the Polish sample) is a one-dimensional tool and consists of 16 items (e.g., “Some groups of people are simply inferior to other groups” or “It’s OK if some groups have more of a chance in life than others”). Participants marked their answers on a seven-point scale ranging from 1 = “Strongly disagree” to 7 = “Strongly agree”.

### Results

Correlation coefficients are presented in Table 4. Confirming our expectations, in the British sample, scores on SDO were correlated negatively with scores on the depression literacy subscale and positively with scores on the misconceptions about depression subscale, with the latter correlation being stronger than the former. Additionally, in the Polish sample, scores on the misconceptions about depression dimension positively correlated with SDO scores. The correlation between depression literacy and SDO, although negative, was not significant. However, the pattern of correlations was similar to that obtained in the British sample, with the correlation between SDO and misconceptions about depression being stronger than the correlations between SDO and depression literacy. These results provide further support for the convergent validity of the DepSter scale’s dimensions’ interpretation, confirming the distinct meaning of its factors, and providing a basis for future studies on this subject.

### Study 9

This study aimed to further test the convergent and divergent validity of DepSter score interpretation by analyzing its relationships with the Big Five personality traits [88].

Personality traits are considered factors related to developing general stigmatizing attitudes [89]. Other studies suggest that low openness and high neuroticism increase prejudice and stereotyping. Furthermore, mental health literacy is associated with higher levels of openness [90]. Further studies supported these results and showed that high openness for experience predicts a low propensity to stigmatize mental disorders and develop stereotypical beliefs about them [70]. Therefore, we hypothesized that scores on the depression literacy dimension would be positively correlated with neuroticism and negatively correlated with openness, and the reverse pattern would be observed with scores on the misconceptions about depression. As depression literacy and misconceptions about depression are not personality traits, we expected these correlations to be relatively low.

### Participants and procedure

Three hundred and sixty-four Polish adults (289 women and 75 men, aged 15–87 years, M = 35.82, SD = 12.20), participated in an online study without compensation. The link to the survey was distributed via multiple web pages, including social media platforms. We did not have specific assumptions concerning the sample size, however, we assumed that we would continue data collection for one week. Such a sample size is large enough to detect a correlation of 0.21 with p = .01 and a power greater than 0.95. The majority of participants had graduated from college (n = 253); however, none of them had majored in psychology or medicine.

The participant’s task was to fill out the DepSter scale and the Ten Item Personality Inventory (TIPI) [91, 92] in randomized order. The internal consistency of the DepSter dimensions was acceptable for the depression literacy subscale (α = 0.65) and high for the misconceptions about depression subscale (α = 0.78).

### Results

As presented in Table 4, in line with our expectations, we observed a negative yet weak correlation between the scores on neuroticism and those on depression literacy, while misconceptions about depression correlated positively. We did not observe significant correlations between depression literacy scores and scores regarding openness to experience. However, we did observe a negative correlation between scores on the misconceptions about depression dimension and this trait. None of the remaining correlations with personality traits were significant.

### Study 10

After establishing the structure and construct validity of DepSter score interpretation and its reliability operationalized as internal consistency, a further aim was to assess the test-retest reliability of the English and Polish versions of the scale. To accomplish this, participants who had previously completed the English version of DepSter were contacted after three weeks and asked to complete it again. Similarly, participants who had previously completed the DepSter scale in its Polish version were contacted three months later.

### Participants and procedure

We calculated our sample size assuming that we wanted it to be large enough to detect a correlation of 0.4 at p = .01 with a power greater than 0.95, which gave us a sample size of 97 participants [56].

One hundred and twelve US participants (54 women, 58 men, aged 20–69 years, M = 37.47, SD = 10.85) filled out an English version of DepSter as a part of a larger study twice with a three-week break via Amazon Mechanical Turk in exchange for $1.60. One hundred and twenty-three participants (99 women, 24 men, aged 15–75 years, M = 31.91, SD = 11.36) filled out the Polish version of DepSter twice with a three-month break between the two measurements as a part of a larger online study without compensation.

### Results

The internal consistency of the English version for misconceptions about depression was high, α = 0.90 for the test and α = 0.92 for the retest, and lower for depression literacy, α = 0.56 and α = 0.65, respectively. The internal consistency of the Polish version of the depression literacy dimension was acceptable for the test, α = 0.66 and retest α = 0.76, and α = 0.73 and α = 0.83, respectively, for misconceptions about depression.

For the English version, the correlation between the test and retest was high for misconceptions about depression dimension, r(112) = 0.87, p < .001, and acceptable for the depression literacy dimension, r(112) = 0.61, all p-values < 0.001. For the Polish version, the correlations were lower, which undoubtedly resulted from a longer period between test and retest, respectively for misconceptions about depression r(123) = 0.76, and for depression literacy r(123) = 0.47, all p-values < 0.001.

These results indicated high test-retest reliability for the English and Polish versions of the DepSter misconceptions about depression subscale and satisfactory reliability for the depression literacy subscale. Overall, these results also attested to the scale’s psychometric adequacy.

### Auxiliary analyses: measurement invariance

As our data come from two language versions of the scale and three different countries, we conducted a measurement invariance analysis using multi-group CFA [93] to assess the psychometric equivalence of DepSter across country groups. First, we evaluated the model with two latent variables separately for participants from Poland (n = 3451), the US (n = 420), and the UK (n = 898) using merged samples from Studies 1–9 and the first wave of Studies 10a and 10b. We then examined the psychometric equivalence of DepSter across the three groups testing: [1] configural invariance, assuming the same factor structure in both groups; [2] metric invariance—additionally assuming equal factor loadings from items to latent variables; and [3] scalar invariance—additionally assuming equal intercepts for the items. We tested invariance using model fit and change in fit indices (i.e., ΔRMSEA, ΔCFI, and ΔTLI). Following Cheung and Rensvold [94] and Vandenberg and Lance [95], we assumed that a change in RMSEA of 0.015 or less and a change in CFI and TLI of 0.01 or less would mean that the two models did not differ; between 0.01 and 0.02 that the two models might have possibly differed; and greater than 0.02 that the two models definitely differed.

For the initial test of the model with two latent variables separately for participants from Poland, the US, and the UK, the maximum likelihood CFA for the Polish and the UK sample yielded an acceptable model fit with respect to most indices, while the model fit indices in the US sample were worse (see Table 6). We concluded that these results provided initial support for configural invariance, and we therefore, conducted a formal test of measurement invariance between participant groups (Table 7).

**Table 6:** Fit indices for the two-factorial model of DepSter tested in three countries

Country	χ^2^/df	RMSEA	90%CI	SRMR	TLI	CFI
PL	17.18	0.068	[0.065, 0.072]	0.032	0.95	0.94
US	5.12	0.099	[0.089, 0.109]	0.083	0.90	0.88
UK	5.06	0.067	[0.061, 0.074]	0.054	0.90	0.88

**Table 7:** Measurement invariance across country groups

Type of invariance	RMSEA	GFI	CFI	TLI	ΔRMSEA	ΔCFI	ΔTLI
Unconstrained model	0.070	0.994	0.942	0.931	-	-	-
Configural	0.071	0.994	0.940	0.928	0.001	0.002	0.003
Metric	0.071	0.993	0.934	0.929	< 0.001	0.006	−0.001
Scalar	0.084	0.991	0.901	0.902	0.013	0.034	0.028
Partial scalar (MiscD items released)	0.080	0.992	0.913	0.909	0.010	0.021	0.020
Partial scalar (MiscD + i4 + i6 released)	0.073	0.993	0.928	0.924	0.002	0.006	0.005

Regarding configural and metric invariance, the ΔRMSEA, ΔCFI, and ΔTLI were below 0.01, indicating that the structure of the scale and factor loadings of latent variables to items did not differ between country groups. However, we found no support for scalar invariance when we imposed constraints on item intercepts, which implied that at least some item intercepts differed between countries. Since we might expect that the misconceptions about depression are grounded in cultural beliefs to a greater extent than depression literacy, we further investigated whether partial scalar invariance could be achieved by releasing constraints on items from this subscale. We found that this resulted in partial scalar invariance with respect to ΔRMSEA, but not to ΔCFI and ΔTFI. We, therefore, continued the backward approach releasing constraints on items from the depression literacy subscale, and found that partially releasing the intercepts for item 4 (“Depression makes people lack the strength to do anything”) and item 6 (“Depression is associated with great suffering”) such that the intercepts were equal for the US and the UK, but different for the Polish sample, resulted in reaching partial scalar invariance.

Overall, we concluded that the measurement model was invariant across country groups with respect to configural and metric invariance. However, we reached only partial scalar invariance for the depression literacy subscale, and we found a lack of scalar invariance for the misconceptions about depression subscale. It is, therefore, possible to make cross-country comparisons concerning the correlation with other variables, although the results of comparison concerning mean scores should be treated with caution due to only partial scalar invariance.

## Discussion

### Contribution to understanding depression literacy

The current work introduces a new approach to measuring beliefs about depression, proposing a two-factorial model and related self-report measure to assess how people vary with regard to depression literacy and beliefs about depression. The paper’s main focus was on developing and validating DepSter, which aimed to be a psychometrically reliable measure for use in further research. In ten studies (total N = 4,688) conducted in three countries, we demonstrated that DepSter is a promising measure of both depression literacy and misconceptions about depression. The two-factorial structure of beliefs about depression was confirmed in four studies conducted on Polish, American, and British samples. In further studies, we established the convergent validity of measurement with DepSter. We found that both a high level of depression literacy and a low level of misconceptions about depression are related to mental health literacy, depression literacy measured with D-Lit, having experience with depression (either being diagnosed with depression or having contact with a person diagnosed with depression), empathetic sensitivity and perspective taking, and emotional stability. This pattern of results confirms that both depression literacy and misconceptions about depression measure some aspects of beliefs about depression. However, we also demonstrated that, although substantially correlated, the two dimensions of beliefs have divergent meanings. Interestingly, having a formal education in psychology differentiated the misconceptions about depression component, but not the depression literacy component. In other words, psychologists may have the same level of evidence-based knowledge concerning depression as non-psychologists. Still, at the same time, they do not incorporate much naïve, stereotypical, and culturally driven information concerning this disorder. This might mean that formal education concerning mental health issues makes people more immune to accepting depression-related information not grounded in scientific evidence. Furthermore, when testing DepSter’s psychometric equivalence across the three groups (Poland, the US, and the UK), we found that the measurement model was invariant across the groups in terms of configural and metric invariance, but not scalar invariance: we achieved partial scalar invariance for the depression literacy subscale, and a lack of scalar invariance for the misconceptions about depression subscale. This latter finding means that people from the three countries scoring equally on the latent variable representing misconceptions about depression have different intercepts on the items from this subscale [96]. This is therefore an indirect confirmation that the misconceptions about depression are to some extent culturally driven.

As expected, we found that the two factors of DepSter have a different relationship to prejudice toward people with mental health illness, depression stigma, personal stigma, social dominance, and openness to experience; the correlations were more robust for misconceptions about depression than for depression literacy. Not surprisingly, basing one’s knowledge on non-confirmed, stereotypical “facts” that lead to a distorted view of people who suffer from depression is mainly connected with the propensity to stigmatize these people. However, we do not know the causal relation between misconceptions about depression and stigmatization. On the one hand, accepting unproven knowledge and formulating a stereotypical view of depression might trigger one’s negative perception of depressed individuals and enhance the propensity to stigmatize them. On the other hand, a high tendency to stigmatize might close one’s mind and trigger confirmation bias when looking for information on depression. According to Nickerson [97], confirmation bias “connotes the seeking or interpreting of evidence in ways that are partial to existing beliefs, expectations, or a hypothesis in hand.” This definition implies that confirmation bias is a purely cognitive phenomenon that amounts to a selective search for information and discrimination in its use. However, confirmation bias might also be seen as part of the broader phenomenon of “motivated reasoning” [98]. For example, research has shown that people engage in “motivated thinking” to defend their beliefs and preserve a positive view of themselves [99]. Thus, holding a prejudice toward people with depression might lead to a selective search for and discriminant use of information about depression, with a preference for information that puts depressed individuals in a negative light. At least to some extent, this reasoning is in line with the fact that misconceptions about depression correlated more strongly with social dominance orientation than did depression literacy. People high in social dominance orientation strain to gain control and power over others [100], especially those they perceive as a threat—this is the core of prejudice toward people with depression.

Our two-factorial model might contribute to explaining why some depression literacy interventions were not effective in reducing the perceived stigma of depression [101]. For example, Griffiths et al. [101] tested the effectiveness of a web-based depression literacy intervention on reducing the stigma associated with depression. They found that the effects of such an intervention on personal stigma were small (Cohen’s d = 0.11) and did not affect perceived stigma. Furthermore, the effects were not mediated by the level of depression literacy measured with D-Lit. We believe that these null results might stem from the fact that the intervention used by the authors provided evidence-based information about depression, including its symptoms, general and specific sources of help, and medical and psychological treatments for depression. The site also indicated that depression is an illness and emphasized that depression can and should be treated as such [29]. However, all this information refers to depression literacy, not to misconceptions about depression which are closely associated with personal stigma. Therefore, even if the intervention affected evidence-based knowledge about depression, it might not attenuate misconceptions about depression that are rooted in cultural beliefs and stereotypes, and hence, not lead to the desired effect on stigma. Accordingly, we see a need for more fine-tuned intervention programs aiming specifically at increasing depression literacy or decreasing misconceptions. Furthermore, it is possible that exposure to evidence-based knowledge might trigger a level of depression literacy only when individuals do not hold a stereotypical view (misconceptions) of depression, i.e., they are open to new, evidence-based information. This proposition should be tested in further empirical programs.

### Limitations and further directions of research

DepSter was designed as a short and easily implementable self-report tool allowing for wide use in research, especially in the social perception of those diagnosed with depression. The fact that DepSter scale is short might have led to the relatively low Cronbach’s α values in some of our studies. However, as the results of test-retest correlations over three weeks and three months are more than satisfactory, we believe that these low α values do not indicate low reliability of the scale, but are rather the result of heterogeneity. Furthermore, self-report measures come with their own limitations, and their use should be supplemented by other, possibly more objective measures when possible. It is also a limitation of the current work that when validating the DepSter scale, its factors relied entirely on self-report measures. Hence, the extent to which the scores on DepSter are associated with actual attitudes, judgments, or preferences is uncertain. A significant research direction for the future is to utilize behavioral measures to test the scale’s construct validity further.

Another limitation of our work is that we tested DepSter in online samples only, altering the way we recruited our participants, including paid online panels such as Polish Ariadna, Prolific Academic, and Amazon Mechanical Turk together with student samples and voluntary recruitment via social media. Although the quality of the data obtained from online labor markets has been questioned, research demonstrates that data collected on Prolific Academic is valid and equivalent to data collected via traditional methods [102–104]. However, it would be interesting to see whether the paper-and-pencil version of the scale has similar psychometric characteristics to its online version.

Although we tested our scale in three different cultural contexts, i.e., Poland, the US, and the UK, the evidence for its two-factorial structure is mixed, with the fit indices suggesting the best fit is in the British sample, followed by the Polish sample, with the American sample providing a worse fit to the bi-dimensional model. We believe that this might be because the American sample was the smallest in size, and we used Amazon MTurk for data collection. Unfortunately, MTurk received some criticism for a decrease in data quality around the time we collected these data [45], and this might have been something that we saw in the result of this study. However, the first Polish sample was relatively homogenous, with a majority of women (82% of the participants). Furthermore, only one of our samples was representative of the society we investigated (Study 2, the Polish sample), we did not preregister our studies, and in most cases, we did not collect detailed information about our participants, such as their ethnicity, education level, socio-economic status, etc. For further preregistered studies, we also wish to collect data from more diverse samples, controlling for additional sociodemographic factors, to allow for better generalization of the results and broader use of the DepSter scale. In particular, we would like to collect data from culturally diverse samples that would be comparable in terms of their representativeness for the respective populations to verify the measurement invariance and potentially allow for cross-cultural comparisons.

Moreover, although we demonstrated that beliefs’ about depression are differentiated by whether the person had contact with people diagnosed with depression or were diagnosed with depression themselves or not, and by the major of education, the cross-sectional design of our studies did not allow for any causal claims. Thus, we would also like to examine the development of beliefs about depression in longitudinal and experimental studies, allowing for such conclusions. For example, it would be interesting to determine whether people who choose to become psychologists have better depression literacy even before starting their education or whether psychological or medical education strengthens their depression literacy. Furthermore, we also wish to examine the short- and long-term predictive value of DepSter and its dimensions for social behavior regarding depressed people and for help-seeking under challenging times.

## Conclusions

Beliefs about depression are a very important topic in mental health research. This importance stems from the fact that this construct is related not only to attitudes toward people who are diagnosed with depression but also to the propensity to stigmatize this mental disorder. In this work, we elaborated on different dimensions of beliefs about depression and introduced the DepSter scale, a 14-item measure of beliefs about depression, which consists of both depression literacy and misconceptions about depression. The initial evidence for the validity and reliability of the scale is very encouraging and suggests that DepSter can be successfully used to measure overall beliefs about depression and can be used by anyone (e.g., researchers, clinicians) interested in understanding the structure, causes, and consequences of depression literacy. We look forward to future research with DepSter and hope that it will contribute to efforts aimed at enhancing depression literacy and hindering misconceptions about depression among the general public.

## Electronic supplementary material

Below is the link to the electronic supplementary material.

Supplementary Material 1: Depression Literacy and Misconceptions Scale (DepSter)

Supplementary Material 2: Skala Przekonań na Temat Depresji (DepSter)

## Data Availability

The datasets supporting the conclusions of this article are available in the Research Box repository, https://researchbox.org/691.

## Footnotes

1 The CFI and TLI are incremental fit indices that compare the theoretical model with a null (independent) model, while RMSEA is an absolute fit index. In our case, RMSEA and SRMR suggested an adequate fit, while CFI and TFI did not, which might imply that the fit for the null model is relatively good. Hence, we investigated the RMSEA for a model that assumed no relation between the 15 items in the DLS scale, and found that it was relatively low, RMSEA = 0.148, 90% CI [0.141, 0.155]. As suggested by Kenny [41] and van Laar and Braeken [42], when the RMSEA of the null model is below 0.158, using incremental fit indices for the evaluation of the proposed model may not be informative.

2 According to the European Qualification Framework [55].

## Abbreviations

ADKQ: Adolescents Depression Knowledge Questionnaire
CFA: Confirmatory Factor Analysis
DepSter: Depression Literacy and Misconceptions Scale
DL: Depression Literacy measured with DepSter
D-Lit: Depression Literacy Questionnaire
DSM: Diagnostic and Statistical Manual of Mental Disorders
HLS-Q6: Health Literacy Survey
ICD: International Classification of Diseases
MHLS: Mental Health Literacy Scale
MiscD: Misconceptions about Depression measured with DepSter
PPMI: Prejudice towards People with Mental Illness
SDO: Social Dominance Orientation
TIPI: Ten Item Personality Inventory
