# Supplementary material for: Depression literacy and misconceptions scale (DepSter): a new two-factorial tool for measuring beliefs about depression
Source: BMC Psychiatry. 2023 May 1;23:300. doi: 10.1186/s12888-023-04796-8 (PMC10150464; doi:10.1186/s12888-023-04796-8)
Supplement: Supplementary file 1 — Supplementary Material 1: Depression Literacy and Misconceptions Scale (DepSter) [file 12888_2023_4796_MOESM1_ESM.docx]

**Appendix**

**Depression Literacy and Misconceptions Scale (DepSter)**

Please Indicate Your Agreement with The Following Statements Using the Scale Below

| 1 | 2 | 3 | 4 | 5 |
| --- | --- | --- | --- | --- |
| Strongly disagree | Disagree | Neither agree or disagree | Agree | Strongly agree |

1. Depression is an illness
2. Depression is just a fad
3. Depression can affect anyone
4. Depression is just a temporary mood deterioration
5. Depression makes people lose interest even in the things they used to enjoy doing
6. Depression affects only the people who are weak and cannot cope with their life
7. Depression makes people lack the strength to do anything
8. To overcome depression, all you need is willpower
9. People with depression often think about suicide
10. To overcome depression, all you need is to get yourself together
11. Depression is associated with great suffering
12. Antidepressant medication start to work right after the intake
13. Depression is just a self-pitty
14. People with depression are mentally weak

**Scoring:**

Depression Literacy (DL): Average of 1, 3, 5, 7, 9, 11

Misconceptions About Depression (MiscD): Average 2, 4, 6, 8, 10, 12, 13, 14
