# Supplementary material for: Depression literacy and misconceptions scale (DepSter): a new two-factorial tool for measuring beliefs about depression
Source: BMC Psychiatry. 2023 May 1;23:300. doi: 10.1186/s12888-023-04796-8 (PMC10150464; doi:10.1186/s12888-023-04796-8)
Supplement: Supplementary file 2 — Supplementary Material 2: Skala Przekonań na Temat Depresji (DepSter) [file 12888_2023_4796_MOESM2_ESM.docx]

**Appendix**

**Skala Przekonań na Temat Depresji (DepSter)**

Używając poniższej skali, zaznacz, do jakiego stopnia zgadzasz się z poniższymi twierdzeniami

| 1 | 2 | 3 | 4 | 5 |
| --- | --- | --- | --- | --- |
| Zdecydowanie się nie zgadzam | Nie zgadzam się | Ani się nie zgadzam, ani się zgadzam | Zgadzam się | Zdecydowanie się zgadzam |

1. Depresja jest chorobą
2. Depresja to zwykła fanaberia
3. Depresja może dotknąć każdego
4. Depresja to tylko chwilowe pogorszenie samopoczucia
5. Depresja sprawia, że ludzie tracą zainteresowanie nawet tymi rzeczami, które kiedyś sprawiały im radość
6. Depresja dotyka tylko ludzi słabych, którzy nie radzą sobie z własnym życiem
7. Depresja sprawia, że ludzie nie mają siły do zrobienia czegokolwiek
8. Wystarczy silna wolna, żeby pozbyć się depresji
9. Osoby, które mają depresję, często myślą o samobójstwie
10. Wystarczy wziąć się w garść, żeby pozbyć się depresji
11. Depresja wiąże się z ogromnym cierpieniem
12. Leki przeciwdepresyjne działają od razu po zażyciu
13. Depresja to użalanie się nad sobą
14. Osoby, które mają depresję, są słabe psychicznie

**Klucz odpowiedzi:**

Depression Literacy (DL): Średnia z 1, 3, 5, 7, 9, 11

Błędne przekonania na temat depresji (MiscD): Średnia z 2, 4, 6, 8, 10, 12, 13, 14
